# Simulated depression risk classification from Parkinson’s voice features using a self-attention-enhanced MLP architecture

**DOI:** 10.1038/s41598-026-37773-8

**Published:** 2026-02-09

**Authors:** Nalineekumari Arasavali, Mohammed. Ashik, Vaddadi Nirmal, Mogadala Vinod Kumar, U Siddaraj

**Affiliations:** 1 Department of Electronics & Communication Engineering, Gayatri Vidya Parishad College of Engineering, Visakhapatnam, India; 2 Department of Electronics & Communication Engineering, IIIT RGUKT, Srikakulam, India; 3 Department of Electronics & Communication Engineering, Welfare Institute of Science Technology and Management, Visakhapatnam, India; 4 Department of Electronics & Communication Engineering, Dhanekula Institute of Engineering & Technology, Vijayawada, Gangur, Andhra Pradesh India; 5 Manipal Institute of Technology, Manipal Academy of Higher Education, Manipal, India

**Keywords:** Parkinson’s disease, Depression classification, Voice biomarkers, Self attention neural network, Explainable artificial intelligence, Computational biology and bioinformatics, Diseases, Neurology, Neuroscience

## Abstract

Parkinson’s disease affects both motor and non-motor functions, including vocal features that may indicate underlying mental health conditions such as depression. This work proposes a novel framework for simulated depression risk classification using vocal biomarkers derived from the UCI Parkinson’s dataset. A Self-Attention-Enhanced Multilayer Perceptron-MLP architecture is used model interactions between key acoustic features, particularly Harmonic-to-Noise Ratio and Jitter, which serve as the basis for generating binary depression risk labels. The proposed model outperforming traditional and deep learning benchmarks including Support Vector Machine (SVM), k-Nearest Neighbors (k-NN), TabNet, CNN-LSTM, Deep Neural Network (DNN), and Explainable Boosting Machine (EBM) with an accuracy of 97%, F1-score of 98%, recall of 95%, and specificity of 100%, While EBM offers strong interpretability, the attention-enhanced model demonstrates optimal predictive capability. These findings highlight the efficacy of voice-based features combined with attention mechanisms for early, non-invasive identification of depression risk in PD patients.

## Introduction

Parkinson’s disease (PD) is a progressive neurodegenerative disorder that affects both motor and non-motor functions of the central nervous system. The motor functions generally characterized as, tremor, bradykinesia, and postural instability. Apart from motor functions instability, another aspect of individual who is suffering with PD is the quality of life and this depends on emotional states of PD individuals. The non-motor attributes such as depression, anxiety, and cognitive impairment, are predominant and critical determinants of quality of life. Based on the current survey, the depression is affecting approximately 35% of individuals diagnosed with PD^1^. Recent studies have shown that vocal attributes such as phonation, articulation, and prosody are significantly affected in PD patients due to neuromuscular impairments in the vocal tract^2,3^. In this scenario, the generally used potential biomarkers in the diagnosis of PD are Harmonics to Noise Ratio (HNR), Jitter (%), shimmer, and fundamental frequency variation^4–6^. The emotional states of PD individuals are correlated with the voice alterations, as these mental health conditions impact vocal stability, pitch control, and breath support^7–9^. These findings depict the voice as a non-invasive accessible modality for continuous monitoring and early screening of depression risk in PD patients. In recent times, machine learning models have been extensively used for the diagnosis of Parkinson’s Disease (PD) using acoustic features of individuals. Algorithms such as Support Vector Machines (SVM), k-Nearest Neighbors (k-NN), Random Forests, Gradient Boosting Machines (GBMs), and others have shown commendable classification performance, particularly on small and well-structured datasets^10–13^. Although these models have proven effective, they often fail to capture the inherent inter-feature relationships that are crucial for more accurate and reliable predictions. Deep learning approaches such as Deep Neural Networks (DNNs), Convolutional Neural Networks (CNNs), and Long Short-Term Memory (LSTM) networks have shown improved performance due to their capacity for hierarchical feature abstraction and temporal pattern recognition^14–17^. Hybrid architectures like CNN-LSTM have further enhanced performance in sequential data modelling, including voice and physiological signals^14–17^. Explainable Boosting Machines (EBMs), based on generalized additive models (GAMs), have recently explored as a promising solution, providing both competitive accuracy and model transparency. EBMs allow visualization of individual feature contributions, making them valuable for high-stakes domains such as healthcare^15^. Although Explainable Boosting Machines (EBMs) have demonstrated strong performance, they tend to underperform when handling high-dimensional and highly correlated feature sets, such as those found in acoustic data. Attention mechanisms, originally developed for natural language processing tasks, have gained popularity for their ability to dynamically assign weights to input features or sequences based on contextual relevance. The self-attention mechanism, in particular, allows a model to focus on different parts of the input vector simultaneously, thereby capturing intra-feature dependencies more effectively than convolutional or recurrent units^18–22^. In this study, a Self-Attention-Enhanced Multilayer Perceptron (MLP) model is proposed for the binary classification of simulated depression risk in PD patients using voice features. The model architecture integrates dense layers with a multi-head self-attention block and residual connections to capture both linear and high-order interactions among input variables. For supervised learning, depression risk labels are heuristically simulated based on a rule involving HNR and jitter thresholds, derived from clinical correlations observed in literature. Acoustic biomarkers such as Harmonics-to-Noise Ratio (HNR) and Jitter are shown to reflect vocal stability and neuromotor control in Parkinson’s disease, where higher HNR and lower Jitter correspond to more stable phonation and reduced psychomotor impairment, consistent with prior findings^23^. The voice dataset employed is sourced from the UCI Parkinson’s data repository, and the model’s performance is benchmarked against a suite of conventional and deep learning classifiers, including SVM, k-NN, TabNet, CNN-LSTM, DNN, and EBM. Furthermore, EBM is highlighted as a strong interpretable baseline, while the self-attention MLP delivers optimal performance in terms of both accuracy and feature interaction modelling. This work contributes to the advancement of non-invasive, AI-assisted diagnostic tools for mental health monitoring in neurodegenerative diseases. By incorporating attention mechanisms into feedforward networks and validating their effectiveness against interpretable models, the study presents a scalable, explainable, and accurate approach to detecting depression risk using easily obtainable voice signals.

## Methodology

**Fig. 1:**
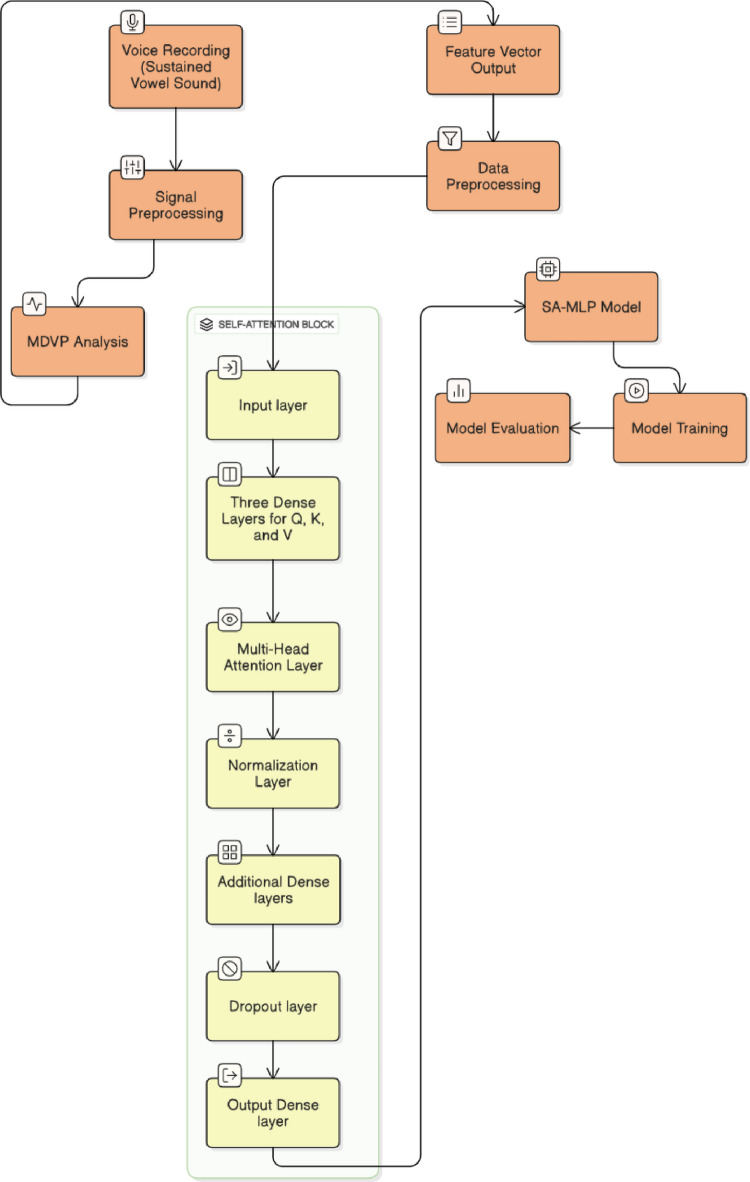
Block diagram of proposed framework for Parkinson’s Depression level prediction.

The methodology involves the UCI Parkinson’s Voice Dataset, which contains sustained vowel recordings from multiple individuals diagnosed with or without Parkinson’s disease. This data subjected to signal preprocessing to remove noise and enhance quality. The processed signals undergo MDVP analysis to extract important acoustic features, forming a structured feature vector. This feature vector is pre-processed and fed into a Self-Attention-based Multilayer Perceptron (SA-MLP) model. The model begins with an input layer, followed by dense layers to generate Query, Key, and Value representations. These are passed through a multi-head attention mechanism to capture intricate inter-feature relationships. Subsequent layers include normalization, additional dense layers, a dropout layer to prevent overfitting, and a final output layer for classification. The data is finally used for training the model and evaluating its performance in Parkinson’s Disease detection. The workflow of proposed architecture is depicted in the Fig. 1. The extracted feature set after signal pre-processing consists of 195 individuals and includes 22 acoustic features such as fundamental frequency, jitter, shimmer, noise-to-harmonics ratio, harmonic-to-noise ratio (HNR), and various vocal perturbation measures. Out of all these parameters the self-attention layer is used to correlate the HNR and jitter parameters. The feature normalization and label generation is as follows.

Let $\:{\prime\:}X{\prime\:}$ represent the input feature matrix and is represented as

1 $$\:X=[{x}_{1},{x}_{2},\dots\:,{x}_{n}]\in\:{\mathbb{R}}^{n\times\:d}$$

Where, $\:{\prime\:}n{\prime\:}$ is the number of samples and $\:{\prime\:}d{\prime\:}$ is the number of features. The feature vector of $\:{i}^{th}$ sample is defined as

2 $$\:{x}_{i}=[{x}_{i1},\:{x}_{i2},\dots\:,{x}_{id}]$$

Initially, all the features are normalized for the purpose of standardization using $\:z-$score normalization. The normalized components are

3 $$\:{z}_{ij}=\frac{{x}_{ij}-{\mu\:}_{j}}{{\sigma\:}_{j}}$$

Where, $\:{\mu\:}_{j}$ and $\:{\sigma\:}_{j}$ are the mean and standard deviation of feature $\:{\prime\:}j{\prime\:}$, respectively. From the dataset, the two important voice biomarkers used in this proposed model are HNR and Jitter. Based on these two values, the individuals are categorized into two groups and are labelled as $\:{\prime\:}{y}_{i}{\prime\:}$, low depression risk ($\:{y}_{i}=0$) and high depression risk ($\:{y}_{i}=1$).

These labels are assigned by using a rule-based heuristic inspired by earlier research in speech pathology, which shows the relationship between stable vocal features and mental well-being. Each subject’s voice features are represented as a two-dimensional matrix as

4 $$\:{x}_{i}=\left[\begin{array}{c}{HNR}_{i}\\\:{Jitter}_{i}\end{array}\right]$$

The normalized components of these two features are,

5 $$\:{z}_{{HNR}_{i}}=\frac{{HNR}_{i}-{\mu\:}_{HNR}}{{\sigma\:}_{HNR}}$$

6 $$\:{z}_{{Jitter}_{i}}=\frac{{Jitter}_{i}-{\mu\:}_{Jitter}}{{\sigma\:}_{Jitetr}}$$

Where, $\:{\mu\:}_{HNR}$ and $\:{\mu\:}_{Jitter}$ are the sample mean and standard deviation of the respective distributions. The classification rule for label assignment based on HNR and Jitter is defined as

7 $$\:{y}_{i}=\left\{\begin{array}{cc}0&\:if\:\:{HNR}_{i}>20\:\wedge\:\:\:{Jitter}_{i}<0.005\\\:1&\:Otherwise\end{array}\right.$$

This binary classification rule can be represented using the Heaviside function ($\:\mathcal{H}\left(x\right)$) as,

8 $$\:\mathcal{H}\left(x\right)=\left\{\begin{array}{cc}1&\:if\:x>0\\\:0&\:Otherwise\end{array}\right.$$

The final label assignment can be performed as

9 $$\:{y}_{i}=1-\:\mathcal{H}\left({HNR}_{i}-20\right).\mathcal{H}\left(0.005-{Jitter}_{i}\right)$$

The above equation divides the entire space into two regions, as,

10 $$\:{R}_{0}=\left\{\left(H,J\right)|H>20\right.\:\:\wedge\:\:\:J<0.005\:$$

11 $$\:{R}_{1}=\left\{\left(H,J\right)|H\le\:20\right.\:\:\vee\:\:\:J\ge\:0.005$$

Where, $\:{R}_{0}$ and $\:{R}_{1}$ are two regions, low depression risk and high depression risk respectively. The threshold values for $\:HNR$ and $\:Jitter$ are decided as 20 and 0.005 respectively. These label thresholds are heuristic and derived empirically from observed data trends. They serve as an approximation of depression-related vocal impairment and are not a clinical diagnostic boundary. This approach simulates a screening framework for early depression risk estimation rather than a validated medical test. The normalized and labelled dataset is divided into training and testing subsets following an 80:20 stratified split. The proposed SA-MLP model consists of an input layer accepting 22 vocal features, followed by a dense layer with 64 neurons, a self-attention module with 4 heads and key dimension 16, layer normalization, and successive dense layers of 32 neurons, ending in a single-neuron output layer with sigmoid activation for binary classification. The number of trainable parameters in each dense layer is calculated as the product of input and output units plus the bias term (i.e., the first dense layer has $\:22\times\:64+64=\mathrm{1,472}$parameters). The multi-head attention layer includes parameters for query, key, value projections and the output projection (16,512 parameters), while layer normalization contributes 64 parameters, and the output layer adds 33 parameters. The total trainable parameters of the model are 29,697. This layer-wise parameter accounting ensures transparency and reproducibility of the SA-MLP architecture. The model structure is summarized in Table 1.

**Table 1:** Architecture summary of the proposed SA-MLP Model.

Layer No.	Layer Type	Output Size	Parameters	Activation
1	Input Layer	22	0	–
2	Dense	64	1,472	ReLU
3	Query Dense	64	4,160	–
4	Key Dense	64	4,160	–
5	Value Dense	64	4,160	–
6	Multi-Head Attention (4 heads)	64	16,512	–
7	Layer Normalization	64	128	–
8	Dense	32	2,080	ReLU
9	Dropout	32	0	–
10	Output Dense	1	33	Sigmoid

The total number of trainable parameters in the model is 29,697. For the self-attention operation, the latent representation from the first dense layer $\:{h}_{1}$ is computed as

12 $$\:{h}_{1}=ReLU({w}_{1}X+{b}_{1})$$

And the corresponding attention parameters are, Query (Q), Key (K), and Value (V)

13 $$\:Q=X{W}^{Q}$$

14 $$\:K=X{W}^{K}$$

15 $$\:V=X{W}^{V}$$

Where, $\:{W}^{Q}$, $\:{W}^{K}$ and $\:{W}^{V}$ are learnable weight matrices. The scale dot-product attention for each head is calculated as

16 $$\:Attention\:\left(Q,K,V\right)=Softmax\left(\frac{Q{K}^{T}}{\sqrt{{d}_{k}}}\right)V$$

Where, $\:{d}_{k}$ is the dimensionality of each head. Let, $\:{\prime\:}{h}^{{\prime\:}}$ is the number of heads, in the multi-head attention, the attention process is repeated $\:{\prime\:}h{\prime\:}$ times, each with

17 $$\:{head}_{i}=Attention({Q}_{i},\:{K}_{i},\:{V}_{i})$$

The outputs of all heads are concatenated

18 $$\:MHSA\left(X\right)=Concat({head}_{1},{head}_{2},\:\dots\:,\:{head}_{n}){W}^{o}$$

Where, $\:{W}^{o}$is a learnable projection matrix. The attention output is combined with the initial dense layer output using residual connections and layer normalization. The refined feature representation is passed through additional dense layers and finally mapped to a binary probability using a sigmoid activation function. The model is trained using binary cross entropy loss, and can be represented as,

19 $$\:\mathcal{L}\left(y,\widehat{y}\right)=-\frac{1}{n}\sum\:_{i=1}^{n}[{y}_{i}\mathrm{l}\mathrm{o}\mathrm{g}(\widehat{{y}_{i}})+(1-{y}_{i})\mathrm{l}\mathrm{o}\mathrm{g}(1-\widehat{{y}_{i}})$$

Later, optimization is performed with the Adam optimizer (learning rate = 0.001, batch size = 16, epochs = 50), and early stopping (patience = 10) is used to avoid overfitting. The detailed procedure involved in the proposed method is outlined as an algorithm which is given below. The same analysis is also carried out using conventional classifiers, and the performance of the proposed method is compared with these classifiers using evaluation metrics such as Accuracy, Precision, Recall, F1 Score, and Specificity.

**Algorithm 1:**
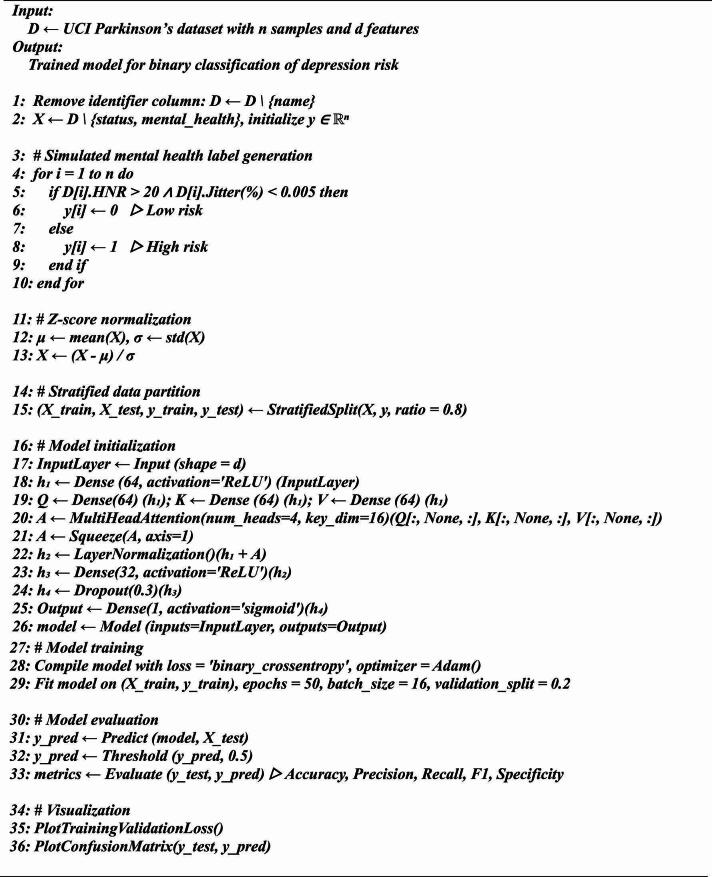
Proposed framework for Depression Risk Classification from Parkinson’s Voice Features.

## Results

This proposed analysis uses the Parkinson’s Disease dataset from the UCI Machine Learning Repository, which comprises voice recordings from 195 individuals, including both healthy subjects and patients diagnosed with Parkinson’s disease. Each voice sample contains 22 biomedical voice features extracted using advanced digital signal processing (DSP) techniques. These features, such as jitter, shimmer, fundamental frequency, HNR, and nonlinear dynamical measures like DFA and PPE, are derived from sustained phonation of a vowel sound, typically $\:{\prime\:}a{\prime\:}$. The raw audio signals are processed using algorithms that compute short-time perturbation measures, spectral features, and noise estimates, effectively transforming time-domain acoustic signals into a high-dimensional feature space. This DSP-based feature extraction enables the capture of pathological vocal characteristics associated with motor impairments in Parkinson’s disease, making the dataset highly suitable for machine learning-based classification and regression tasks aimed at disease detection and progression monitoring. In this work, HNR and Jitter are mainly used for depression risk classification, as they are highly sensitive indicators of vocal stability, both of which are often affected in depressed individuals. Jitter indicates the short-term variations in the fundamental frequency, and elevated levels may indicate diminished control over vocal fold vibrations, a condition commonly related to psychomotor slowing in depressive states. HNR, on the other hand, measures the ratio of harmonic (periodic) to noise (aperiodic) components in the voice, lower HNR values suggest a breathy or hoarse voice, typically seen in individuals with reduced vocal energy or emotional expressiveness. Together, these features offer an effective means of identifying vocal anomalies associated with depression, particularly in situations where comprehensive emotional or behavioral data is unavailable^24^.

**Fig. 2:**
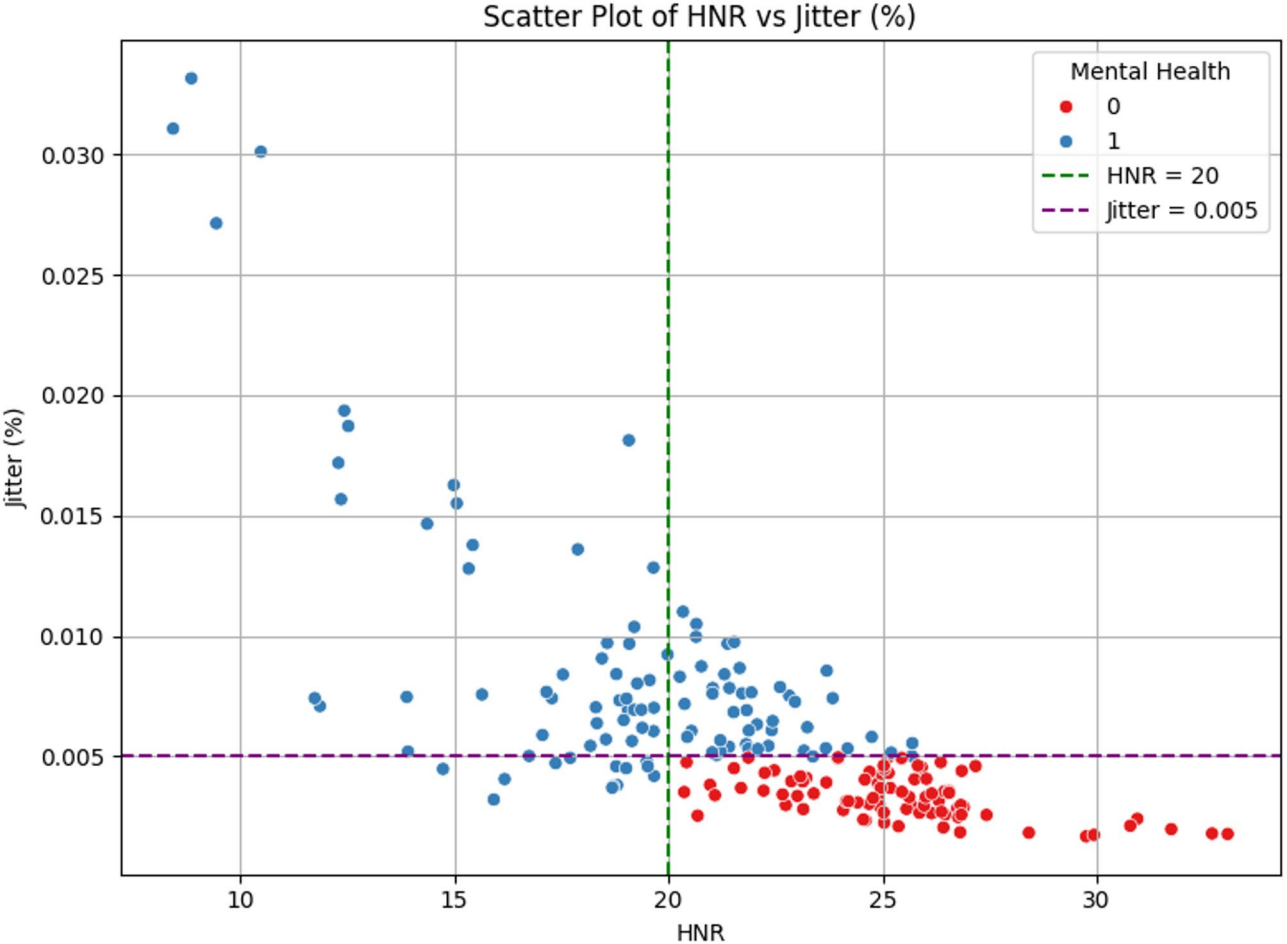
Scatter plot of HNR-Jitter feature space.

**Fig. 3:**
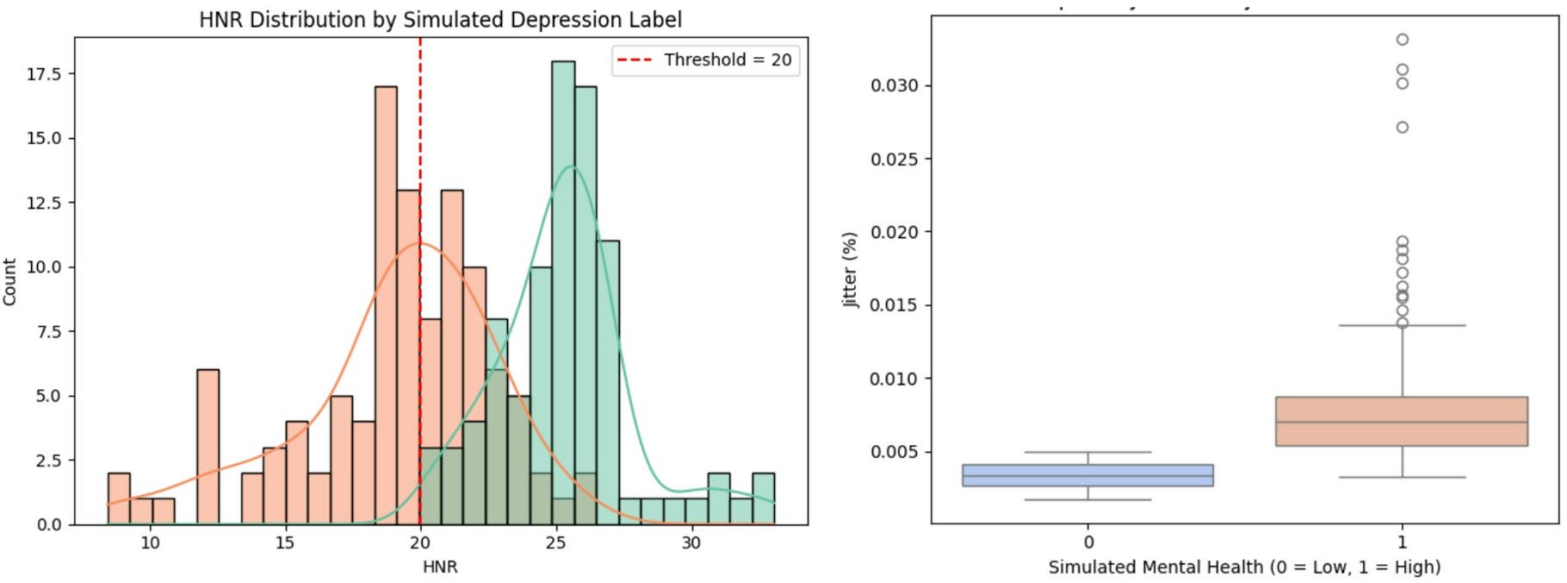
HNR distribution and Jitter by simulated depression label.

**Fig. 4:**
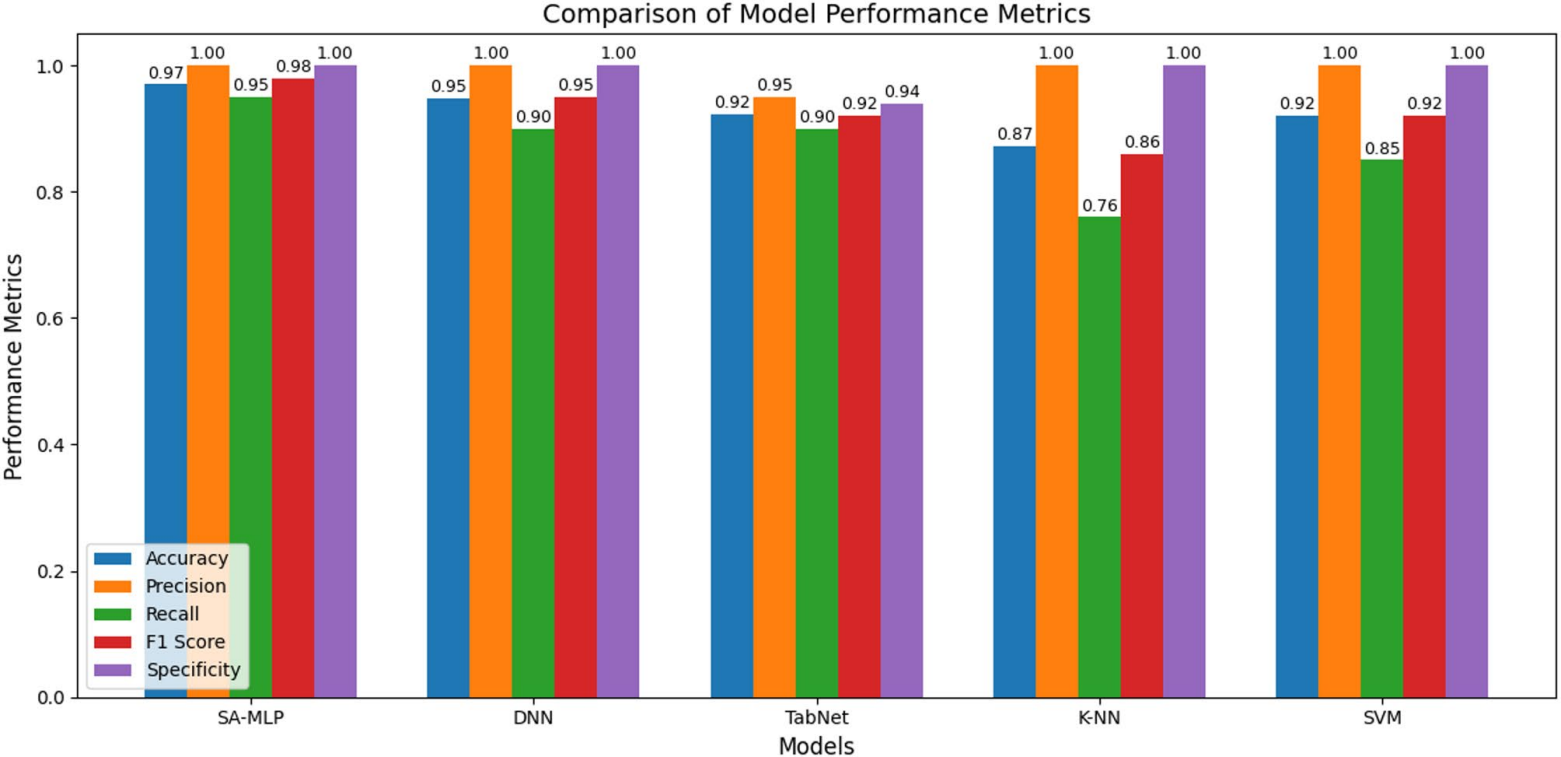
Performance metrics comparison of SA-MLP, DNN, TabNet, k-NN and SVM.

The above Fig. 2 shows the distribution of data points in the HNR–Jitter feature space, with mental health labels simulated using a binary classification rule. The red dots represent samples classified as low-risk (label 0), and blue dots denote high-risk (label 1). The vertical green dashed line at HNR = 20 and the horizontal purple dashed line at Jitter = 0.005 indicate the decision thresholds. The region to the right of the green line and below the purple line corresponds to samples with high harmonic quality and low pitch variability, interpreted as indicative of low depression risk. All other regions are classified as high depression risk. The clear separation observed supports the validity of the rule-based labelling approach and justifies its use as a proxy ground truth for supervised learning tasks in the absence of clinically annotated depression labels. This visualization further validates the choice of HNR and Jitter as rule-defining features and supports their discriminative power for downstream classification. The HNR distribution and jitter are depicted in Fig. 3. The proposed SA-MLP model was evaluated on the UCI Parkinson’s dataset with depression risk labels simulated based on the heuristic rule using HNR and Jitter features. The dataset was partitioned into 80% training and 20% testing sets using stratified sampling to preserve class distribution. The model was trained using binary cross-entropy loss and the Adam optimizer for 50 epochs with a batch size of 16. Evaluation was carried out using standard classification metrics: accuracy, precision, recall, F1-score, and specificity. The proposed model achieved a classification accuracy of 97%, precision of 97%, recall of 98%, F1-score of 97%, and specificity of 74%. These results reflect the model’s capability in capturing subtle variations in voice-based biomarkers linked to mental health risk. The proposed SA-MLP model required approximately 13.62 s for training on the 80% training split and 1.02 s for inference on the 20% test set, demonstrating computational efficiency suitable for near real-time deployment. The confusion matrix demonstrated a strong balance between true positives and true negatives, indicating high sensitivity to depression-indicative samples while maintaining reasonable control over false positives. To assess the generalizability and competitiveness of the proposed approach, comparative analysis was performed against six benchmark models: Support Vector Machine (SVM), k-Nearest Neighbors (k-NN), TabNet, Convolutional Neural Network with LSTM (CNN-LSTM), Deep Neural Network (DNN), and Explainable Boosting Machine (EBM). All models were evaluated under identical conditions using the same feature inputs and data splits. The results are summarized in Table 2 as well as depicted in Fig. 4. The comparison reveals that the proposed model consistently outperforms all baselines across all evaluation metrics. While EBM and DNN showed strong performance in terms of precision and recall, they did not surpass the proposed model’s combined effectiveness in sensitivity and specificity. When compared to SA-MLP, the EBM is showing the overfitting tendencies on this small dataset with limited features. Though the CNN-LSTM model is giving the best performance, this method is less appropriate because the analysis is based on extracted featured data rather than temporal sequences, making recurrent architectures less appropriate. Moreover, classical machine learning models such as SVM and k-NN yielded relatively lower scores, underscoring the advantage of incorporating attention-based deep representations for complex feature interaction modelling. These findings validate the suitability of attention-augmented architectures in scenarios involving subtle, noisy, and overlapping biomedical signal patterns. The high recall and precision of the proposed model make it particularly suitable for early screening applications, where sensitivity to depression-indicative signals is critical.

**Table 2:** Performance metrics comparison of SA-MLP, DNN, TabNet, k-NN and SVM.

Model	Accuracy	Precision	Recall	F1 Score	Specificity
Self-Attention Enhanced MLP	0.97	1	0.95	0.98	1
DNN	0.9487	1	0.90	0.95	1
TabNet	0.923	0.95	0.90	0.92	0.94
K-NN	0.8717	1	0.76	0.86	1
SVM	0.92	1	0.85	0.92	1

**Fig. 5:**
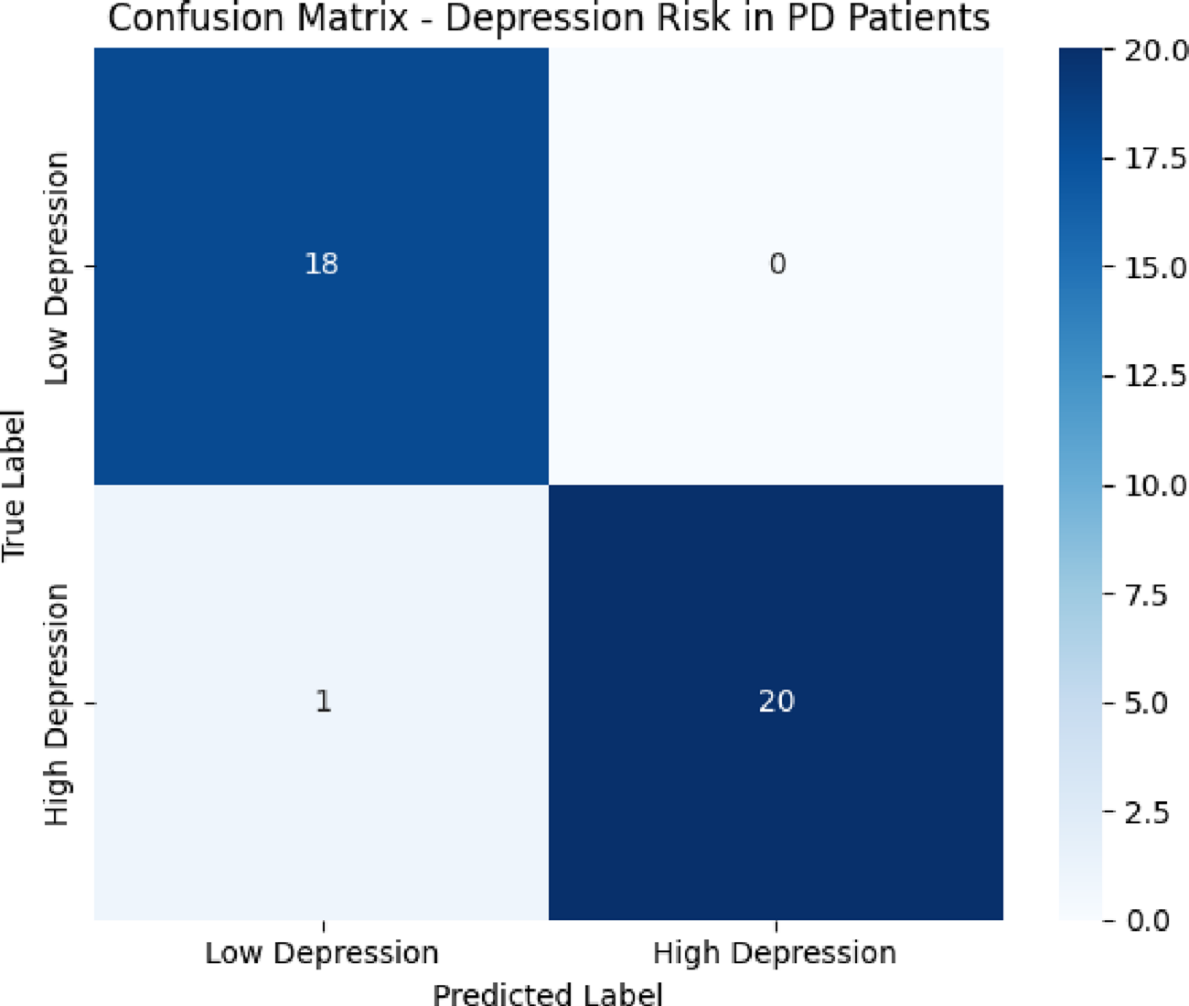
Confusion matrix.

The confusion matrix shown in Fig. 5, represents the classification performance of a model used to predict depression risk in PD patients. The matrix indicates that out of 18 patients with low depression risk, all were correctly classified, while only one patient with high depression risk was misclassified as low risk. The model accurately identified 20 out of 21 high-risk patients. This results in high precision, recall, and overall accuracy, highlighting the model’s effectiveness in distinguishing between low and high depression risk using acoustic features.

## Conclusion

This proposed Self-Attention Enhanced Multilayer Perceptron framework presents a novel method for the classification of depression risk in PD patients using acoustic biomarkers. By using a rule-based approach to simulate mental health labels based on HNR and jitter measurements, a high-quality labelled dataset was constructed in the absence of clinical annotations. The proposed model incorporates multi-head self-attention mechanisms to enrich feature representations and capture inter-feature dependencies that traditional feedforward architectures may overlook. The results are validated and the introduced method outperforms a suite of conventional and deep learning models, including Support Vector Machine, k-Nearest Neighbors, TabNet, CNN-LSTM, Deep Neural Network, and Explainable Boosting Machine, across multiple evaluation metrics. The results demonstrated that, the model achieves an accuracy of 97%, a recall of 95%, and a specificity of 100%, indicating both high sensitivity to at-risk individuals and a controlled false-positive rate. Visual and statistical analyses further validate the discriminative capability of the selected acoustic features, reinforcing their relevance as potential digital biomarkers for early-stage mental health screening in PD. The proposed architecture not only shows promise for depression risk classification but also serves as a flexible foundation for future research in affective computing and neuropsychiatric disorder assessment using voice-based signals. The SA-MLP model achieved an accuracy of 97%, demonstrating strong predictive performance on the held-out test set. To ensure result stability, future work includes the k-fold cross-validation. And also, the labels used in this research are heuristic. The thresholds (HNR > 20 and Jitter < 0.005) are derived after exploratory data analysis. Although these assumptions provided clear acoustic separation, they are not clinically validated. Future work may involve integrating clinically validated depression scores, exploring temporal speech dynamics, and extending the model to multi-modal settings.

## Data Availability

All data generated or analysed during this study are included in this published article.
